# Laminarin Induces Defense Responses and Efficiently Controls Olive Leaf Spot Disease in Olive

**DOI:** 10.3390/molecules26041043

**Published:** 2021-02-17

**Authors:** George T. Tziros, Anastasios Samaras, George S. Karaoglanidis

**Affiliations:** Laboratory of Plant Pathology, Faculty of Agriculture, Forestry and Natural Environment, Aristotle University of Thessaloniki, POB 269, 54124 Thessaloniki, Greece; gtziros@yahoo.gr (G.T.T.); samarasanast@gmail.com (A.S.)

**Keywords:** *Fusicladium oleagineum*, systemic acquired resistance, *Olea europaea*, phenylalanine ammonia lyase, plant resistance-inducers

## Abstract

Olive leaf spot (OLS) caused by *Fusicladium* *oleagineum* is mainly controlled using copper fungicides. However, the replacement of copper-based products with eco-friendly alternatives is a priority. The use of plant resistance-inducers (PRIs) or biological control agents (BCAs) could contribute in this direction. In this study we investigated the potential use of three PRIs (laminarin, acibenzolar-S-methyl, harpin) and a BCA (*Bacillus amyloliquefaciens* FZB24) for the management of OLS. The tested products provided control efficacy higher than 68%. In most cases, dual applications provided higher (*p* < 0.05) control efficacies compared to that achieved by single applications. The highest control efficacy of 100% was achieved by laminarin. Expression analysis of the selected genes by RT-qPCR revealed different kinetics of induction. In laminarin-treated plants, for most of the tested genes a higher induction rate (*p* < 0.05) was observed at 3 days post application. *Pal, Lox, Cuao* and *Mpol* were the genes with the higher inductions in laminarin-treated and artificially inoculated plants. The results of this study are expected to contribute towards a better understanding of PRIs in olive culture and the optimization of OLS control, while they provide evidence for potential contributions in the reduction of copper accumulation in the environment.

## 1. Introduction

Olive (*Olea europaea* L.) is the emblematic tree of the Mediterranean Basin, as it is a plant species well-adapted to the unique environmental conditions prevailing in the surrounding countries [1]. Over the 70% of the globally cultivated olive trees are located in the European Union’s Mediterranean countries, with Greece being the third producer country in the world with an average annual production of 300,000 Mg olive oil, following Spain and Italy [2]. The regular consumption of olive oil, in the frame of the Mediterranean diet, is related with several beneficial effects on human health [3]. For instance, it provides protection against cardiovascular diseases and chronic diseases, such as cancer, inflammatory and neurodegenerative diseases [4].

Olive leaf spot (OLS), a foliar disease also known as peacock spot or bird’s eye spot, is caused by the biotroph fungal pathogen *Fusicladium oleagineum* (syn. *Spilocaea oleaginea*, *Cycloconium oleagineum*), according to the recently proposed use of the Genus *Fusicladium* instead of *Venturia* for those species which present only anamorphic stage [5]. It is one of the most important fungal diseases that affect olive trees, and in cases of severe infections could cause yield losses of approximately 20% [6]. The disease causes distinctive lesions mainly on the upper surfaces of the leaves, which are initially inconspicuous sooty blotches, but later develop into muddy green to almost black circular spots surrounded by a yellow halo [4]. Petioles, fruits and stems are also susceptible, but rarely display lesions [7]. The infected leaves fall prematurely, and defoliation affects the vegetative and reproductive growth of olive trees in a negative way [6].

OLS is mainly controlled by chemical fungicides, usually those that contain copper (Cu), such as Bordeaux mixture, copper hydroxide, copper oxide and copper oxychlorides [8]. In olive-growing regions, which are characterized by long dry summers, OLS is controlled by the application of copper-based products before winter rains and directly after harvest [5]. Nonetheless, the timing of fungicide applications is crucial for the effective control of the disease [6,9]. Copper-based fungicides usually control OLS in cases of low disease incidence, no matter what is the product applied, the application rate or the number of applications [7]. Thus, regular annual applications are required in order to prevent disease development and possible severe disease levels that may be difficult to control [9]. 

The innate plant defense against a broad range of microorganisms such as fungi, oomycetes, bacteria and viruses is a process known as induced resistance (IR) [10]. IR is divided into systemic acquired resistance (SAR) and induced systemic resistance (ISR), which generally vary in the signaling pathways and molecules through which local and systemic defense are acquired [11]. SAR is induced after localized exposure to a pathogen, or after treatment with synthetic or natural compounds, and is related to the accumulation of salicylic acid (SA) and the activation of non-expressor of pathogenesis-related protein 1 (NPR1) [12,13]. On the contrary, ISR is a response induced by plant growth-promoting rhizobacteria (PGPR) or compounds such as antibiotics, surfactants or other chemicals [14]. ISR in not associated with the accumulation of SA, but is dependent on jasmonic acid (JA) and ethylene (ET) signaling pathways [15]. In addition, plant resistance can be induced via the application of plant resistance-inducers (PRIs), such as chemical compounds, plant or microbe extracts, or non-pathogenic microbes such as plant growth-promoting rhizobacteria or fungi [16,17].

During the last few decades, several chemical compounds or plant and microbial extracts have been registered for use in several crops as PRIs, also known as plant resistance activators, plant defense activators, or elicitors. They have a broad target spectrum, although factors such as plant genotype, stage of growth, environmental conditions, timing and way of application may affect their performance against plant pathogens [17]. 

Acibenzolar-S-methyl (ASM) is a plant activator which induces SAR and, subsequently, protection against a large number of plant pathogens, including *F. oleagineum* [8,11]. SAR, induced by acibenzolar-S-methyl, is accompanied by an increased level of salicylic acid (SA), locally as well as systemically, and by the up-regulation of a specific set of genes encoding PR proteins (PRs), which are supposed to lead to disease resistance [10]. The linear β-1,3-glucan laminarin, a polysaccharide extracted from the brown algae *Laminaria digitata*, has been reported as an efficient plant resistance-inducer in various plant species [18]. On the other hand, harpins are glycine-rich and heat-stable proteins that are secreted through the type III secretion system in Gram-negative plant-pathogenic bacteria [19]. *Bacillus* species reveal antagonistic activities that are associated with the production of metabolites with antibiotic properties. *Bacillus*-based biological control agents (BCAs) have been used to control various plant parasitic microorganisms as they are able to reproduce actively and to withstand unfavorable environmental conditions [20,21,22]. 

Taking into account that olive crop is heavily treated with copper fungicides, Cu-minimizing measures are a priority in reducing the risk for environmental damage imposed by Cu-accumulation. Among the measures that could contribute to the reduction in Cu-use in olive orchards environments is the replacement of Cu or other chemical fungicides by PRIs. Furthermore, *Pseudomonas* and *Bacillus* strains were screened for their efficacy against OLS only under in vitro conditions [23]. 

The current study was conducted aiming to a) determine the efficacy of three commercial PRI products and one biological agent against OLS on young olive plants under greenhouse conditions, and b) to provide further insights into the molecular mechanism associated with the induction of olive plants’ resistance to OLS via laminarin treatments. 

## 2. Results

### 2.1. Disease Assessment and Control Efficacy

**Figure 1:**
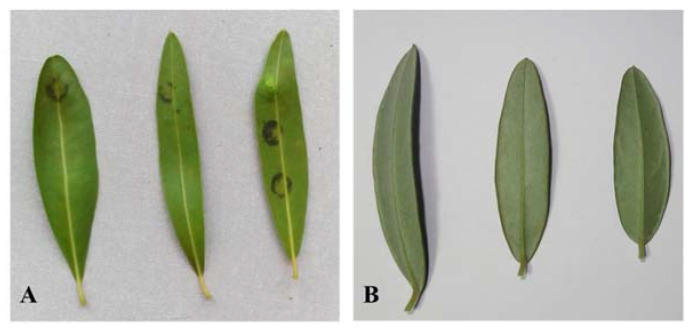
Olive leaf spot (OLS) lesions appeared on olive leaves after immersion in sodium hydroxide. (**A**) Leaves from control plants. (**B**) Leaves from laminarin-treated plants.

**Figure 2:**
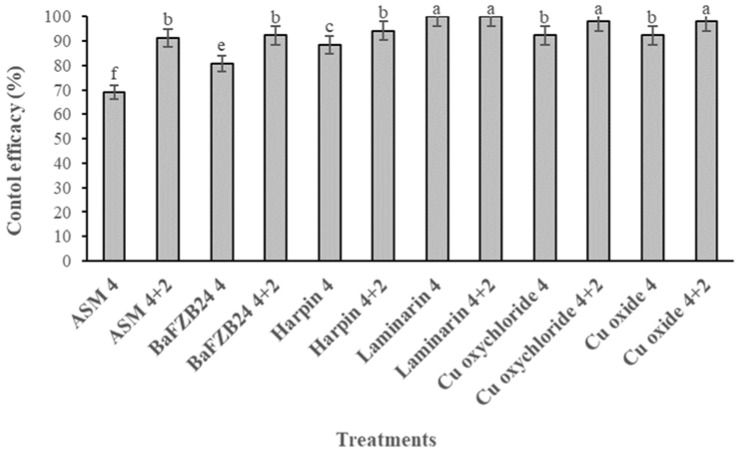
Control efficacy (%) of olive leaf spot achieved by several resistance-inducers, a biological control agent or copper fungicide treatments applied either in a single application 4 weeks before inoculation or in a dual application 4+2 weeks before the inoculation with *Fusicladium oleagineum*. Each value is the mean of three replicates ± standard error. Different letters on the columns indicate significant differences among treatments according to Tukey’s multiple range test at *p* = 0.05. Vertical lines indicate the standard error of the mean.

Artificial inoculations with the *F. oleagineum* isolate used in the study were successful. Disease symptoms started to appear on control plants four weeks after the inoculation. As expected, the highest disease severity was observed on untreated control plants. Disease severity was significantly lower compared to that on control plants in all the treatments independently, whether they had been applied in a single or a dual application, while, interestingly, no symptoms were observed on plants treated with laminarin, either in a single or in a dual application (Figure 1). For the remaining treatments, the control efficacy achieved by the dual applications was always higher compared to the respective efficacy values achieved by the single applications conducted 4 weeks before the inoculation of the plants (Figure 2). Thus, laminarin ensured the higher control efficacy values of 100% when applied either as a single or a dual treatment (Figure 2). A similarly (*p* < 0.05) high control efficacy against OLS was achieved by the two conventional copper products, but only when they had been applied in dual applications 4 and 2 weeks before the inoculations. In contrast, the single application of the two conventional copper fungicides resulted in a control efficacy lower than that of laminarin application (Figure 2). All the remaining treatments provided control efficacy values significantly lower than that of laminarin. The lower control efficacy value of 68% was achieved by the single application of acibenzolar-S-methyl 4 weeks prior to the inoculation (Figure 2).

### 2.2. Defense-Genes Expression in Laminarin-Treated Non-Inoculated Plants

**Figure 3:**
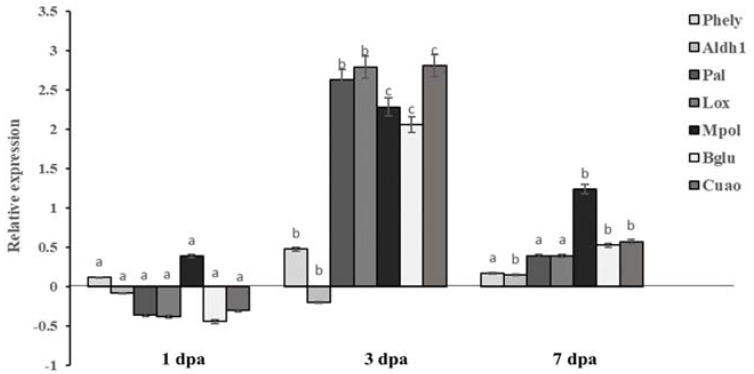
Expression analysis of *Olea europaea* defense-associated genes by real-time quantitative PCR (RT-qPCR) at three different time-points after application of laminarin (1 dpa, 3 dpa and 7 dpa). The y-axis represents fold differences in gene expression compared to that of plants before laminarin application (time point 0 h). Actin gene was used as endogenous control. Each value is the mean of three biological and three technical replicates ± standard error. Different letters on the columns indicate significant differences inside each gene studied for the three time-points according to analysis of variance (ANOVA) at *p* = 0.05.

Increased expression levels of some target genes (alcohol dehydrogenase (*Aldh1*), phenylalanine ammonia-lyase (*Phely*), 9-Lipoxygenase (*Lox*), major pollen allergen (*Mpol*), Beta-1,3-glucanase (*Bglu*), copper amine oxidase (*Cuao*), phenylalanine ammonia-lyase *(Pal*)) were observed at all time points in laminarin-treated as compared to non-treated plants at time point 0h, confirming that this treatment may trigger defense responses in olive. Maximum induction was measured three days after the application for all genes except *Aldh1* (Figure 3). The expression levels showed different patterns at different time points. For instance, only two genes (*Phely* and *Mpol*) were over-expressed at the early time point (1 dpa), while the remaining five tested genes were down-regulated. At 3 days post-application, all but *Aldh1* genes tested were found to be up-regulated, and for most of them the whole relative expression was increased more than twofold compared to the time point 0 h (Figure 3). Similarly, at the last assessed time point (7 days post-application), a transcription induction level for all genes was observed. Among the seven tested genes, the *Mpol* gene showed the higher expression level; however, for all but the *Aldh1* genes tested, the expression levels were lower than those observed at 3 dpa (Figure 3).

### 2.3. Defense-Genes’ Expression in Laminarin-Treated and Inoculated Plants

**Figure 4:**
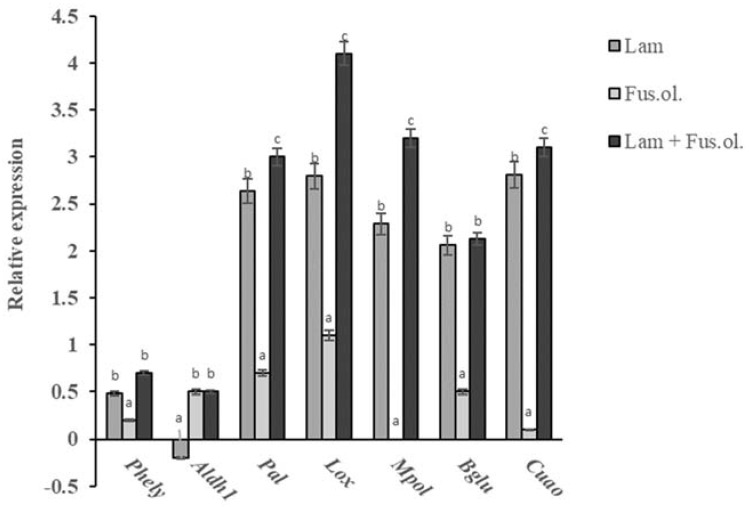
Expression of defense-related genes in olive plants treated with laminarin (Lam), artificially inoculated with *Fusicladium oleagineum* (Fus. ol.), treated with laminarin and artificially inoculated plants with *F. oleagineum* (Lam + Fus. ol.). Transcription levels were determined by real-time quantitative PCR (RT-qPCR) 1 day after inoculation with *F. oleagineum* or 3 days after laminarin application. Results were expressed as the fold increase in transcript levels and normalized to mock-inoculated plants. The actin gene was used as endogenous control. Values represent the mean of triplicates of the experiment. Different letters on the columns indicate significant differences inside each gene studied for the three different treatments according to analysis of variance (ANOVA) at *p* = 0.05.

Based on the findings of gene expression analysis in laminarin-treated plants, a multi-treatment experiment was conducted to incorporate the measurement of gene expression in plants treated with laminarin and/or inoculated with the pathogen. Gene expression data are showed in Figure 4. The treatment of plants with water did not change significantly the expression of any tested gene. Artificial inoculation with the pathogen changed the transcription levels of *Lox*, causing a one-fold increase, while for the remaining genes tested their expression levels were only slightly increased (Figure 4). In contrast, the laminarin treatment caused a higher than twofold increase in *Pal, Lox Mpol, Bglu* and *Cuao*, while a slight increase of only 0.5-fold was observed for *Phely* (Figure 4). Higher induction levels for all tested genes but the *Aldh1* were observed in olive plants that had received both laminarin treatment and artificial inoculation with the pathogen. In these plants, *Lox* was induced at a rate higher than four-fold compared to the untreated mock-inoculated plants. Similarly, induction levels higher than three-fold were observed for *Pal, Cuao* and *Mpol* (Figure 4).

## 3. Discussion

Olive crop is one of the most heavily treated crops with copper fungicides, since a fairly high number of copper spray applications is required during spring and autumn periods to successfully control major foliar diseases, such as OLS, or fruit diseases, such as anthracnose [9]. However, this leads to an increased risk of the accumulation of high copper concentrations in the olive orchard environment, and in particular olive groves soil [24]. Despite the widely accepted need for a reduction in Cu accumulation in the olive groves environment, research related to the development of methods or means that could enable the achievement of this target is limited [8,25]. Taking into account that the reduction of soil contamination by heavy metals is a priority, in the current study the effects of some resistance-inducers and one BCA in controlling OLS were evaluated under controlled conditions.

PRIs have the advantage of being more environmentally friendly, exhibiting reduced negative effects on humans and other living organisms [17]. Moreover, numerous PRIs provide a wide resistance, which subsequently limits the development of resistant pathogen strains, and thereafter could be included in integrated pest management (IPM) programs, prolonging the effectiveness of chemical pesticides [17].

The influence of various factors, including pathogen inoculum concentration, temperature, wetness duration, leaf age and incubation conditions, on OLS development was evaluated under controlled conditions [26]. In that study, although plants exhibited the same level of infection whether they were kept in a growth chamber or in a shadehouse, the disease severity was lower in plants incubated in the growth chamber, as many infections remained latent. Under the experimental conditions of our study, all the tested products were proven effective against OLS. Both the PRIs and the BCA tested were more effective when they were applied in dual applications four and two weeks prior to inoculation with the pathogen. This is in accordance with previous findings of a study aiming to determine the control efficacy of systemic acquired resistance-inducers against OLS [8]. Among the resistance-inducers tested, laminarin was proven to be the most effective. Laminarin reduced the disease severity in the same way, whether it was applied once (4 weeks) or twice (4+2 weeks) prior to pathogen inoculation. For instance, Salah et al. [27] reported that laminarin reduced the mortality of olive twigs inoculated with *Verticillium dahliae* by 20% compared to the untreated plants. Laminarin also effectively reduced *Botrytis cinerea* and *Plasmopara viticola* on grapevine [28]. Furthermore, foliar pre-treatment of a susceptible grapevine cultivar with laminarin reduced the development of *P. viticola* and disease severity when applied on leaves at three different application rates [29]. To the best of our knowledge, this is the first report on laminarin efficacy against this major disease of the olive. However, further studies under field conditions are required to ensure its high efficacy against the disease under the variable environmental conditions prevailing in the olive culture regions.

The remaining PRIs tested were less effective compared to laminarin. However, their efficacy was significantly higher when olive plants received dual applications with them. In a previous study, acibenzolar-S-methyl significantly reduced OLS severity by an average of 48–68% compared to the untreated control [8]. Numerous previous studies have shown that ASM is a potent inhibitor of diseases caused by both fungal and bacterial pathogens on several hosts [30,31,32]. The increased resistance of ASM-treated plants has been associated with a higher activation rate of principal antioxidant enzymes, such as peroxidase, superoxide dismutase, catalase and ascorbate peroxidase, an enhancement of polygalacturonase-inhibiting proteins, and the increased production of PR-proteins [13].

Harpin was the second most effective product against OLS, among the PRIs tested. Similar ranges of disease control have been reported for the species closely related to *F. oleagineum*, *Venturia inaequalis* and *V. pyrina*, causal agents of apple and pear scab, respectively [33]. However, in the same study the sterol demethylation inhibitor penconazole provided greater protection against apple and pear scab in comparison to the plant inducers. A similar effect was also shown in our study, in which the two copper-based fungicides were more effective against OLS compared to harpin.

Various *Bacillus* species have been identified as plant-growth promoting bacteria and/or biocontrol agents [34]. Among them the most studied species are *B. amyloliquefaciens*, *B. licheniformis*, and *B. subtilis,* which are able to enhance plant growth and to trigger specific defense-related pathways, such as induced systemic resistance (ISR), against diseases [35,36]. *Ba* FZB24 is one of the most extensively studied biocontrol agents registered for use against several diseases on numerous hosts [37]. However, to the best of our knowledge this is the first report on the control of an olive disease using *Ba* FZB24. However, the investigation of *Ba* FZB24’s performance against OLS under field conditions is crucial for further use, since it is well established that the efficacy of BCAs may be differentiated in the field [38,39].

The resistance of olive to OLS has been associated with both physical and chemical factors. Among the physical factors, trichome density and cuticle thickness have been recognized as the most important [40], while among chemical parameters, phenolic compounds are those determining the resistance of olive to OLS [41]. Olive leaf and olive fruit extracts are dominated by a vast variety of phenolic compounds, such as oleuropeine, rutin, tyrosol and others, with some of them exhibiting strong antifungal properties [42]. Some of these phenols, such as oleuropein and rutin, have been associated with the induced resistance of olive to OLS [41].

To obtain insights into the molecular mechanisms associated with the increased efficacy of laminarin treatments against OLS, in our study the expressions of seven genes known to be involved in defense were analyzed using RT-qPCR in plants that had been treated with laminarin. The selected genes were encoding the following: phenylalanine ammonia-lyase (*Pal*), a key enzyme of the phenylpropanoid pathway [43]; 9-lipoxygenase (*Lox*), an enzyme of the octadecanoid pathway [29]; copper amine oxidase (*Cuao*) implicated in H_2_O_2_ production [44]; alcohol dehydrogenase (*Aldh1*), which is involved in the biosynthesis of the phenolic portion of secoiridoids and other related phenolic compounds [45]; beta-1,3-glucosidase (*Bglu*), which is involved in phenolic degradation playing an important role in the formation of oleuropein and ligstroside derivatives [46]; and major pollen allergen (*Mpol*), which belongs to the 1,3-glucanases that have been described as pathogenesis-related proteins because of their induction by pathogens [47]. Laminarin stimulates defense responses in cell suspensions of tobacco [48], grapevine [28] and alfalfa [49]. In these studies, several defense responses were reported, such as the activation of mitogen-activated protein kinases, Ca^2+^ influx, oxidative burst, and alkalinization of the extracellular medium.

RT-qPCR data of our study showed that, in laminarin-treated plants, all but the *Aldh1* genes showed their maximum transcript levels three days post-application. At this time point, the higher ratios of induction were obtained for *Pal*, *Lox* and *Cuao.* In a previous study, laminarin application in *Arabidopsis* plants manifested the induction of the LOX1 gene, involved in the synthesis of oxylipin compounds such as JA [50]. Although seven days after laminarin application, all genes were still up-regulated compared to 0 dpa, transcription levels were lower compared to those observed at 3 dpa, except for *Aldh1*, which started to up-regulate at that time point. In grapevine, the induction of defense-related genes by laminarin was found to be much faster (5h post-application), suggesting that the host plays a key role in the activation of these mechanisms [28].

In laminarin-untreated but artificially inoculated plants, the expression levels of the tested genes were slightly altered in contrast to the findings of Benitez et al. [51], who had reported an extensive reprogramming of expression in genes involved both in primary and secondary metabolism following the inoculation of olive with *F. oleagineum*. Such differences are possibly due to the fact that in our study, the expression analysis of the tested genes in artificially inoculated plants was conducted at only one time point, 24 h after the inoculation. Interestingly, the higher induction rates for all but the *Aldh1* genes were observed in plants that had received a treatment with laminarin, and had been artificially inoculated with the pathogen. In these plants, a greater than three-fold increase in expression level was observed for *Pal*, *Lox*, *Mpol,* and *Cuao*. This pattern indicates that the specific proteins most likely do not form part of pathogen-related pathways, but they are involved in different metabolic pathways that induce defense mechanisms.

Several previous studies have shown that laminarin is an effective elicitor of early signaling events, which include the regulation of cytocolic [Ca^2+^] variations, H_2_O_2_ production, plasma membrane depolarization and MAPK activation [52]. Such signals, in turn, lead to the induction of defense-related genes encoding the synthesis of pathogenesis-related (PR) proteins, such as chitinases or glucanases, antimicrobial compounds of phenolic origin, such as phytoalexins, or compounds associated with cell-wall reinforcement [28,48,52]. *Pal*, *Lox*, *Mpol*, and *Cuao* were the genes, among those tested, with the higher induction rates in laminarin-treated and artificially inoculated olive plants. *Pal* is the primary enzyme of the phenylpropanoid pathway that plays a crucial role in phenolic compounds and SA biosynthesis [43,53]. The increased expression of *Pal* observed in both inoculated and non-inoculated laminarin-treated plants is in agreement with findings of previous studies suggesting that laminarin treatments induced *Pal* in grapevine, tobacco or tea plants [28,48,54]. Lipoxygenases (*Lox*) are enzymes that catalyze the production of oxylipins, which are among the signaling molecules of plant immune responses to plant pathogens [55]. Enhanced *Lox* expression following laminarin treatments has previously been reported on tobacco and grapevine plants [28,48]. H_2_O_2_ production is one more mechanism associated with the resistance responses of plants following laminarin treatments. In our study, *Cuao* was found to be highly expressed in laminarin-treated olive plants. *Cuaos* are major partners in polyamine homeostasis in plants [56]. They participate in polyamine oxidation, which in turn leads to H_2_O_2_ generation and the increased resistance of plants to abiotic and biotic stress through the hypersensitive response (HR) [57]. HR is a major resistance mechanism primarily against biotroph pathogens such as *F. oleagineum* [5]. In addition to its contribution in H_2_O_2_ generation, *Cuao* has been shown to participate in the biosynthesis of phenolic compounds in olive [44], thus its increased expression may lead to the increased resistance of olive plants through a double way. The last gene found to be overexpressed in laminarin-treated olive plants was *Mpol*. *Mpol* encodes in olive a PR-protein-exhibiting 1,3-β-glucanase activity [58]. Pathogenesis-related (PR) proteins are among the most widespread allergen proteins associated with plants, and they are, currently, organized into 17 distinct families. PR proteins are mainly induced by plant pathogens, but in addition they can be synthesized in response to abiotic factors [59]. Increased expression rates, following laminarin treatments, of genes encoding glucanases and chitinases, the two major groups of PR-proteins, have been previously reported in grapevine [33,35] and in tobacco [48].

In conclusion, in the current study, three different resistance-inducers and one biological control agent were evaluated for their efficacy against OLS disease under controlled environmental conditions. The control efficacy that they provided was higher than, or at least similar to, that of conventional copper products used as reference treatments. Among the evaluated products, laminarin was found to be the most effective. Gene expression analysis in plants treated with laminarin and infected or not with *F. oleagineum* revealed that the application of laminarin induced a significant increase in defense-related genes, such as *Pal*, *Lox* or *Cuao*. The increased expression of these genes may account for the optimum performance of laminarin treatments against the disease. Thus, laminarin application could reshape OLS control in olive culture by replacing traditional copper fungicides, and in this way, it could contribute to the reduction of copper accumulation in the environment. However, further research is required to determine the efficacy of these products under field conditions, since it is well established that environmental parameters such as the temperature, the light or the relative humidity account for differences in olive tree tolerance to OLS under field and laboratory conditions.

## 4. Materials and Methods

### 4.1. Plant Material

The experiments were carried out on six-month-old olive plants of the susceptible cultivar “Chalkidikis”. The plants were derived from disease-free olive cuttings grown in a commercial nursery, specialized in the production of olive plants. Olive plants were grown in individual pots containing soil and maintained in a greenhouse at 22 ± 3 °C and 50–60% RH until they were used.

### 4.2. Product Applications and Artificial Inoculations

Three different PRIs (acibenzolar-S-methyl, laminarin and harpin) and one BCA (*B. amyloliquefaciens* FZB24) were evaluated against *F. oleagineum*. Two copper formulations (copper oxide and copper oxychloride) registered for use against the pathogen were used as reference treatments. A complete list of the evaluated products is provided in Table 1. They were applied preventively either in a single application 4 weeks prior to the artificial inoculation of the plants or in dual application 4 and 2 weeks prior to inoculation. Applications were conducted with a hand sprayer to run off (approximately 20 mL of spraying solution was used per plant). Control plants were treated with sterile water. Ten replicate plants were used per treatment, and the experiment was repeated three times. After the applications, the plants were returned to the greenhouse until pathogen inoculation, arranged in a completely randomized design.

A single-spore isolate of *F. oleagineum* belonging in the fungal collection of the Plant Pathology Lab, AUTh, was grown on olive leaf extract medium for 5 days at 18 °C [60]. The fungus was cultured in a liquid medium containing 5 g glucose in 500 mL of olive leaf extract, prepared by boiling 20 g of healthy leaves in 1 L of distilled water for 20 min. The medium was then autoclaved for 20 min before use. The produced conidia were harvested in sterile distilled water and inoculum suspension was adjusted at a concentration of 5 × 10^4^ conidia/mL. The inoculum suspension was applied onto the olive plants using an atomizer until just before runoff. Thereafter, the plants were covered with polyethylene bags for 48 h in the greenhouse to maintain high RH, and thus provide sufficient conditions for the infection. In the greenhouse the mean daily temperature was kept at 18 ± 2 °C.

### 4.3. Disease Assessment

The assessment of disease symptoms presence was initiated four weeks post inoculation and continued at weekly intervals until the 12th week post-inoculation. At the 12th week after inoculation, when new spots were not supposed likely to develop, 10 randomly selected leaves per plant were removed and checked for the development of OLS. The measurement of spot number per plant had been proven before to provide a reliable method for the estimation of disease severity [61].

The percentage of infections on leaves was estimated following the sodium hydroxide method [62]. To reveal the latent infections developed on leaves, the leaves were immersed in a 5% NaOH solution for 30 min at room temperature (22 ± 2 °C). Following this treatment, the visible lesions were more distinguishable, and at the same time the latent infections appeared as black circular spots or rings, differentiated in this unambiguous way from the surrounding healthy green tissue. The control efficacy of each treatment was calculated as the percentage reduction in spot numbers compared to the control treatment.

### 4.4. RNA Extraction and Defense-Related Gene Expression in Laminarin-Treated Olive Plants

After completing the determination of the evaluated products’ efficacy against OLS, laminarin was found to be the most effective PRI. For this reason, the expressions of seven defense-related genes (alcohol dehydrogenase (*Aldh1*), phenylalanine ammonia-lyase (*Phely*), 9-Lipoxygenase (*Lox*), major pollen allergen (*Mpol*), Beta-1,3-glucanase (*Bglu*), copper amine oxidase (*Cuao*), phenylalanine ammonia-lyase *(Pal*)) in olive plants were investigated in laminarin-treated plants in comparison to plants treated with water. Samples were collected 1, 3 and 7 days post-application of laminarin (hereafter 1 dpa, 3 dpa and 7 dpa, respectively).

In addition, a multi-treatment experiment was conducted to determine the relative gene expression patterns in: (a) untreated plants artificially inoculated with *F. oleagineum,* (b) laminarin-treated and artificially-inoculated plants, (c) laminarin-treated and mock-inoculated plants, and (d) untreated and mock-inoculated plants. Based on the results derived from the gene expression measurements in the first set of experiments (laminarin-treated plants), tissue samples for RNA extraction were obtained 3 dpa. Laminarin applications were conducted 48 h before inoculation with *F. oleagineum* and the samples were collected 1 day after inoculation with the pathogen. The collected leaf material was immersed, immediately after its removal from the plants, in liquid nitrogen and stored afterwards at −80 °C until it was used for further analysis. For each treatment and respective time point, three plants were used and the whole experiment was repeated three times.

### 4.5. RNA Preparation

For RNA analysis, each sample was composed of three biological replicates (RNA pooled) and three technical replicates per treatment. The collected leaves were ground to a fine powder using liquid nitrogen and stored at −80 °C until use. Thereafter, total RNA was extracted from 250 mg of tissue using the Nucleo Spin RNA Plant kit (Macherey-Nagel, GmbH & Co. KG, Düren, Germany) according to the manufacturer′s instructions. The concentration of the extracted RNA was measured using a P330 nanophotometer (Implen GmbH, Munich, Germany).

### 4.6. Quantification of Gene Expression Levels with RT-qPCR

Total RNA, extracted as described above, was used as a template for RT-qPCR. The 7 genes selected and the primers used are listed in Table 2. The RT-qPCR reactions were performed using a StepOne Plus Real-Time PCR System (Applied Biosystems, Waltham, MA, USA) using a SYBR Green based kit (Luna Universal One-Step RT-qPCR Kit, New English Biolabs, Ipswich, MA, USA) according to the manufacturer′s instructions. The amplification conditions were 55 °C for 10 min, 95 °C for 2 min, followed by 40 cycles of 95 °C for 10 s and 60 °C for 1 min, while the melt curve stage consisted of 95 °C for 15 s, 60 °C for 1 min and 95 °C for 15 s. The threshold cycle (Ct) was determined using the default threshold settings. The 2^−ΔΔCt^ method was applied to calculate the relative gene expression levels [63]. The actin gene was used as the endogenous control and gene expression levels were normalized with laminarin-treated plants at time point 0 d. For the multi-treatment gene expression experiment, samples were normalized with untreated/mock-inoculated plants.

### 4.7. Data Analysis

Disease severity values for all treatments were transformed to percent control efficacy values based on disease severity on the untreated control plants. All data for the three replicate experiments were combined and subjected to analysis of variance (ANOVA) to evaluate the effect of the different treatments, time of application and their interactions. Percentage values were arcsine transformed before statistical analysis. Analysis of variance was performed with SPSS v25.0 (SPSS Inc., Chicago, IL, USA). Significant differences were determined using Tukey’s multiple range test at the *p* < 0.05 level. The significance level of all hypothesis testing procedures was predetermined at α = 0.05. Diagrams were constructed using Graphpad Prism 7.0.

## Figures and Tables

**Table 1:** Products evaluated in this study for their efficacy against olive leaf spot disease.

Active Ingredient	Commercial Name	Concentration Dose (L^−1^ Water) ^a^	Supplier
acibenzolar-S-methyl	BION 50WG	100 mg	Syngenta
*Bacillus amyloquefaciens* FZB24	Taegro 13WP	2.11 g	Syngenta
laminarin	Vacciplant 4.5SL	1 mL	Arysta
harpin	Proact WDG	0.1 g	Κ&Ν Efthymiadis
copper oxychloride	Cupravit 50WP	2.6 g	Κ&Ν Efthymiadis
copper oxide	Nordox 75WG	1.7 g	Κ&Ν Efthymiadis

^a^ application doses were the commercially recommended rates for each product.

**Table 2:** Primer sequences used for gene expression analysis in real-time quantitative polymerase chain reaction (RT-qPCR) assays.

Primer Name	Sequence (5′-3′)	Size (bp)	Gene	Reference	Accession Number
**OePAL-F**	AATGGGGAGCTTCATCCATCA	155	Phenylalanine ammonia-lyase (*Pal*)	[45]	JX266200
**OePAL-R**	AGAAATGTGGATGACATAAGCTTCA				
**OeCUAO-F**	AAGATGGCCTTGGGAAGAAT	191	Copper amine oxidase (*Cuao*)	[45]	GQ851613
**OeCUAO-R**	TTCTGCCAATCCTGTTCTCC				
**OeALDH1-F**	TTTAAGTGGGGAGCTCAAATACA	200	Putative alcohol dehydrogenase (*Aldh1*)	[45]	JX266197
**OeALDH1-R**	GATGCTTCAGATATTCCCATGC				
**BGLU-F**	TTTCACGCGTTGGTAATCCG	180	Beta-1,3-glucanase (*Bglu*)	This study	AJ810085.1
**BGLU-R**	CAGCCTTTTCAAGTGCTGCA				
**Mpol-F**	TGTTCCCCAACCTCCAGTTT	186	Major pollen allergen (*Mpol*)	This study	XM_023036359.1
**Mpol-R**	TCCTTCTGCTCTCGTGTAACC				
**LOX-F**	CAAGCGAAACACCAGAACCA	180	9-Lipoxygenase (*Lox*)	This study	EU678670.1
**LOX-R**	CCACGGATCCTCCAAGAACC				
**OlPhely-F**	CAAAAGCCTAAACAAGATCG	188	Phenylalanine ammonia-lyase (*Phely*)	This study	XM_023030332.1
**OlPhely-R**	CAGGGGTGGCTTGAAAATTC				
**OlActin-F**	GAGCGGGAAATTGTGAGAGA	195	Actin (*actin*)	This study	AF545569
**OlActin-R**	CTGGTAAAGAACCTCAGGAC				

## Data Availability

The data presented in this study are available in this article.

## Sample Availability

Not available.
